# Developing a digital informed consent app: opportunities and challenges of a new format to inform and obtain consent in public health research

**DOI:** 10.1186/s12910-023-00974-1

**Published:** 2023-11-08

**Authors:** Luuk V. Haring, Joy T. Hall, Anton Janssen, J. Marleen Johannes, Arnoud P. Verhoeff, Joanne K. Ujcic-Voortman

**Affiliations:** 1 Department of Healthy Living, GGD Amsterdam, Amsterdam, the Netherlands; 2 Department of Youth Health Care, GGD Amsterdam, Amsterdam, the Netherlands; 3 Sarphati Amsterdam: Research for Healthy Living, Amsterdam, the Netherlands; 4 Faculty of Social and Behavioural Sciences, University of Amsterdam, Amsterdam, the Netherlands

**Keywords:** Parental consent, Multimedia, Electronic consent, Public health research, User experience

## Abstract

**Background:**

Informed consent procedures for large population-based cohort studies should be comprehensive and easy-to-use. This is particularly challenging when participants from different socio-economic groups and multicultural ethnic backgrounds are involved. Recently, more and more studies have tried to use multimedia in informed consent procedures. We describe the development and testing of a digital informed consent app and elaborate on whether this may contribute to a comprehensive and practical procedure to obtain informed consent for public health research.

**Methods:**

In a sample of parents with young children, we used a mixed method approach to study the user experience of an informed consent app and evaluate whether it can be used to adequately inform people and register their consent. Through semi-structured interviews we investigated participants’ experiences with and opinions about the app, with a special focus on comprehensibility of the content and the usability of the app. Information retention questions were asked to evaluate to what extent participants could recall key aspects of the provided study information.

**Results:**

The 30 participants in this study used the app between 4 and 15 min to give their consent. Overall, they found the app well-designed, informative and easy to use. To learn more about the study for which informed consent is asked, most of the participants chose to watch the animated film, which was generally found to convey information in a clear manner. The identification process was met with mixed reactions, with some feeling it as a secure way to give consent, while for others it contradicted their view of using data anonymously. Information retention questions showed that while all participants remembered various aspects of the study, fewer than half answered all four questions satisfactorily.

**Conclusion:**

Our study shows that a well-designed informed consent app can be an effective tool to inform eligible participants and to record consents. Still, some issues remain, including trust barriers towards the identification procedure and lack of information retention in some participants. When implementing consent procedures that incorporate digital formats, it may be beneficial to also invest in a complementary face-to-face recruitment approach.

**Supplementary Information:**

The online version contains supplementary material available at 10.1186/s12910-023-00974-1.

## Background

With over 170 nationalities, Amsterdam is one of the most multicultural cities in the world. More than half of its inhabitants are of non-Dutch descent [1]. The city is also diverse in terms of educational level, with 30% of the population having a university degree, but also 16% of the population having low literacy skills i.e. language skills, math skills and digital skills [2, 3]. Conducting population-based health research in such a diverse population, especially when informed consent is required, is a challenge. In order to obtain a substantially large and representative sample of the population, one needs to consider the differences in capacities and needs of people when developing an informed consent procedure. An informed consent procedure ideally ensures that potential participants understand the research and voluntarily decide to participate. As part of this process, participants learn about study procedures, potential risks and benefits of participation, and their rights. It is therefore essential that the provided information is comprehensible and informative for all potential participants, regardless of socio-economic position, cultural or ethnic background or literacy skills. Numerous studies, however, report limitations of informed consent forms when it comes to properly informing participants [4–6]. Particularly for individuals with low incomes or low levels of literacy, there are indications that informed consent procedures frequently fail to truly inform them sufficiently [7, 8]. The risk of selective non-response due to informed consent procedures in public health studies can seriously reduce the external validity of findings [9]. In recent years, various studies looked for ways to improve informed consent procedures, including modifying procedures or forms or developing procedures that utilize multimedia incorporating video, graphics and audio. In particular, the use of digital consent forms with multimedia has recently been a popular research topic [10–15]. Digital formats allow for remote consent collection, making it particularly suitable for large-scale public health research as it is a cost-effective and sustainable way to engage large numbers of participants. In recent years many studies reported positive effects of the use of multimedia either in terms of improved comprehension of the study, better information retention or increased participant satisfaction [10, 12, 16]. Nevertheless, an improved comprehension through the use of multimedia is not found in every study [13, 14]. This may in part relate to the quality of the multimedia used. In order to successfully develop a new effective informed consent procedure, several researchers argue for the structural involvement of experts and lay stakeholders i.e. the target audience in the process of development [17–19]. The Sarphati Cohort is a large population multi-ethnic dynamic cohort study among children in Amsterdam, the Netherlands. Before the start of the inclusion for this cohort study, we developed a digital informed consent procedure which uses multimedia to register parental consent. This app, by means of an animated video and complimentary text, makes the information accessible for parents with various social and cultural backgrounds. By making the app multilingual i.e. English, French, German, Spanish and Turkish, we also aim to reach parents with limited Dutch language proficiency in groups that comprise a significant proportion of the Amsterdam population. With this effort, we aim to ensure that all parents can make a well informed decision.

The version of the informed consent app that was tested in this study was developed in several stages. Throughout these stages of development i.e. from a paper print version to a fully functional app, we involved several stakeholders, including the end users: parents. The focus of this qualitative study was to investigate whether the developed informed consent app can contribute to a comprehensive and practical procedure for informed consent. The main research questions are (1) what is the opinion of parents on the usability, look & feel and content of the informed consent app? and (2) how well did the informed consent app inform parents about the study?

## Methods

### Study design

This qualitative study with a mixed-method design included a usability and content test, information retention questions and an in-depth semi-structured interview to obtain parental feedback on an informed consent app. All were done consecutively within one session.

### Setting and recruitment

This study was conducted in preparation of implementing the informed consent procedure for the Sarphati Cohort. The cohort systematically monitors growth and its determinants in Amsterdam children from birth until adulthood (18 years of age). Data collection within the Sarphati Cohort is linked to routine consultations with Youth Health Care. In addition to this core set of data, data are collected through age-specific questionnaires covering a wide variety of health related topics (e.g. sleep, nutrition, physical activity, screen usage, home environment, parenting experiences).

In order to obtain a diverse sample of Amsterdam parents we recruited participants (n = 30) by convenience sampling at various locations, such as public libraries, market squares, playgrounds and Youth Healthcare Centres in multiple neighbourhoods in Amsterdam. In addition, we recruited participants from a group that had previously participated in a study on child health questionnaires. People were included if they met all of the following criteria: (1) at least 18 years of age; (2) parent of a young child, preferably between 0 and 4 years old; (3) resident of Amsterdam; (4) able to read and understand Dutch sufficiently; (5) did not have extensive prior knowledge of the Sarphati Cohort. Parents who were interested in participation were contacted to schedule an appointment at the location of their choice. Most test sessions took place at the participants’ homes. Two sessions took place at the participant’s request at their work and one session was held at our office.

### Participants

Thirty participants of whom 5 males and 25 females took part in this study. Twenty-nine parents had at least one child between the ages of zero and four. The remaining parent had 3 children, the youngest of whom was five years old. Nearly a third of the participants exclusively identified themselves with a non-Dutch cultural group or identified themselves with a second cultural group in addition to Dutch (i.e. 2 Turkish, 2 Surinamese, 1 Moroccan, 1 Polish, 1 Mexican and 1 Finnish). 21 participants completed higher education (i.e. university or college degree), 7 participants had completed intermediate levels of education (i.e. intermediate secondary education) and 2 participants had only completed basic levels of education (i.e. not achieving beyond lower vocational or technical secondary education).

### Materials

**Fig. 1:**
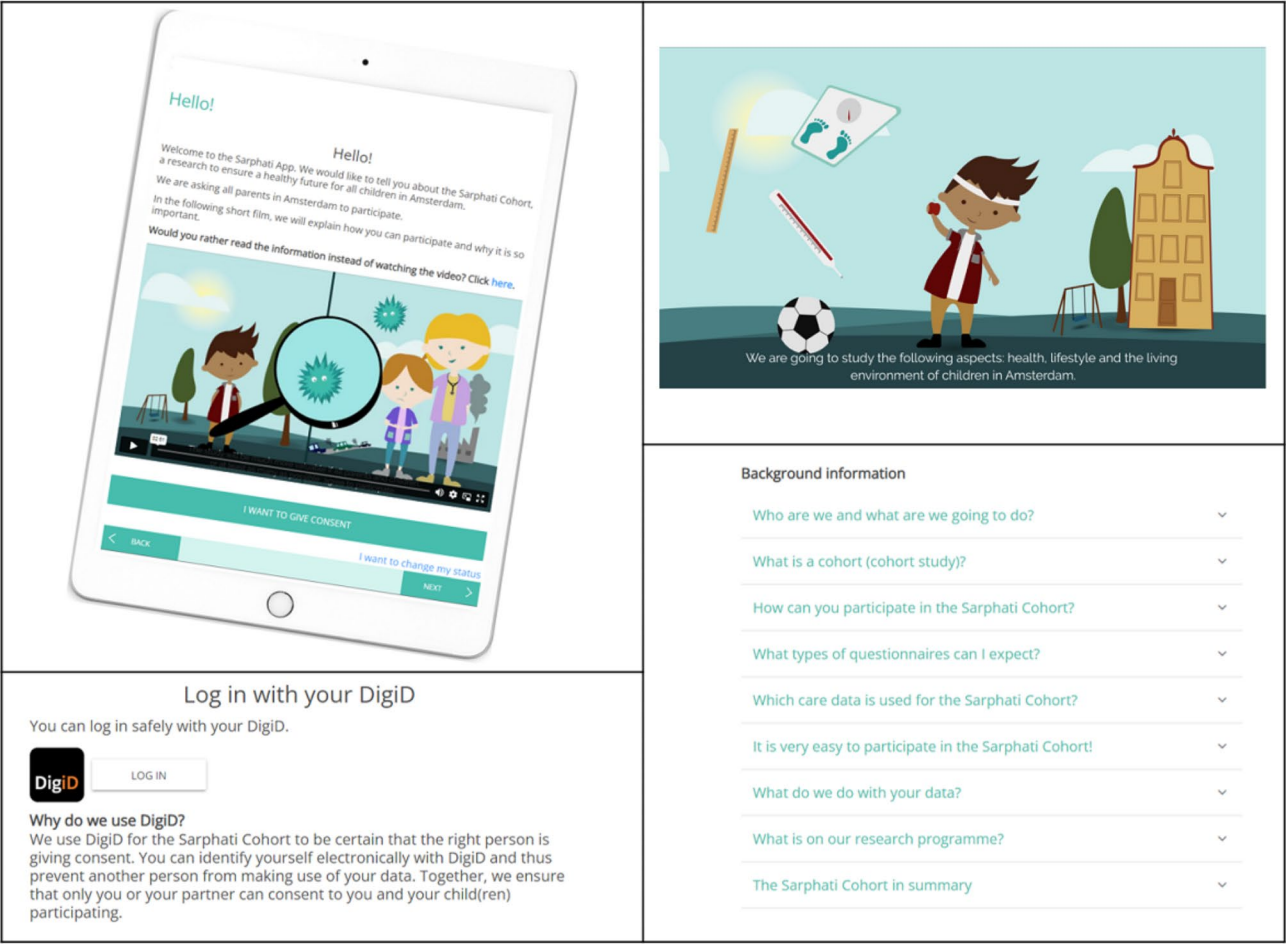
Screenshots of the English version of the Sarphati App (released in 2018)

Participants were provided with a 9,7” iPad (6th gen) device on which the app was accessible via the Safari web browser (http://sarphati-app.nl). The prototype web-based version of the app was only available in Dutch. The app contained: a three-minute explanatory animated film, the script in text form; supplementary text blocks with detailed background information; a digital identification procedure containing the Dutch governmental identification tool DigiD. DigiD allows individuals to identify themselves when making arrangements on the internet. For instance with local governments, health care institutions or pension funds. Only organizations that are legally authorized to use citizen service numbers and meet strict requirements are allowed to use DigiD as an identification tool. This ensures that their personal data are always protected [20]. As the cohort study records parental consent in their child’s medical records, a strict identification measure like DigiD is necessary. This enables us to verify that a person is eligible to participate and is allowed to give parental consent; a page to fill out the consent form; and a menu through which parents can adjust their information, withdraw their consent or view the information again (Fig. 1). Participants also received a note with the information required (DigiD username & password and date of birth of the child) to give consent for the test children.

### Procedure

The test sessions were conducted between March and April 2018. Prior to the test participants signed an informed consent and agreed to the sessions being audio recorded. To get an impression of our sample of participants, we first noted some background characteristics of the participants such as gender, age, ethnic identification, education, family situation and the age of their children. After the test, participants received a 10-euro gift voucher.

### Usability and content test

We asked participants to go through the app, log in via a mock-up DigiD page and give consent for one or more children. We gave the participants a note with fake log-in credentials designed for this test and necessary info of the corresponding dummy children. For the sake of the test, we asked the participants to consider these children as if they were their own. As well, to get a realistic picture of how people might interact with our app we purposely did not ask the participants to thoroughly study all the content provided in the app. As such, participants were free to choose the type and amount of information they deemed necessary for their decision to participate. While testing the app, using a think-aloud method, we encouraged participants to verbalize their thoughts about the content and functionality of the app.

### Information retention questions

After completing the usability and content test, participants were asked several questions about the content of the study. This was done to gain insight into the extent participants had understood the provided information and could reproduce it. We asked what they thought the study entailed, what was asked from participants, what type of information of their child would be used and how the data of their child would be handled.

### Semi-structured interview

Lastly, a semi-structured interview was held to further discuss parents’ experiences with and views on the app. Topics included: (1) overall experience of the informed consent app, including look & feel, usability, and content; (2) desired improvements for the app; (3) the quality of the provided information i.e. animation and texts; and (4) opinion on the identification procedure (i.e. DigiD sign-in).

### Analysis

Audio recordings of the sessions were transcribed by the researchers following a clean read approach [21]. Relevant characteristics and topics from the transcripts were traced after repeated reading of the interviews. These were then listed into a coding framework. To ensure the reliability of the coding framework, three researchers coded the transcripts in MAXQDA in alternating pairs independently of each other. The coded fragments were then compared by the researchers. Discrepancies found between codes were discussed and agreed upon to settle uniformity. Analyses of the information retention questions were done in a similar manner. Answers were assessed and coded by two researchers on correctness and to what extent participants had fully answered the question. Any differences between codes were discussed and agreed upon.

## Results

### Overview of results

Participants spent between 4 and 15 min, with an average of 8 min, using the app to inform themselves about the study and give their consent. With the exception of one participant, who encountered a technical problem in the identification procedure, all could successfully follow the steps to give consent for the study. Nearly all participants mentioned they found the app well-designed and for most parts user friendly. Some participants pointed out that two pages where not user friendly due to buttons on unexpected places and entry fields that were not displayed properly. The look and feel of the app, i.e. including page layout and use of colour, was said to be clear and appealing.

### Themes

After repeated reading of the interviews, the following themes were considered to be the most important by the participants: (1) time required to give consent; (2) opinions on the app’s content and preferences for types of information sources; (3) opinions on the identification procedure. A fourth theme (4) on participants’ ability to recall study information; was included to better understand how the app performed as an informative tool.

#### Time required to give consent

Participants collected information in various ways to gain knowledge about the Sarphati Cohort. The majority of participants used the animated video as their sole source of information. Others, in addition to the film, read some of the background information text items. Two participants exclusively used texts to inform themselves about the study. Six participants stopped halfway through the video, some of whom, before continuing, took a brief look at the supplementary text items. They commented that a 3-minute video was too long and that it should either be shortened or divided into smaller parts. They felt that some of the information provided, in particular on data protection, was too detailed and suggested making those parts optional. Some argued their rationale for skipping information was that if they had made the effort to download the app, it likely would have meant they were already aware of the study and would have the intention to participate.

*I stopped the video. It’s nice that the app doesn’t contain too much information. The information is easy to read. A video of 3 min is too long. You decide to participate beforehand. So what is the purpose of the video? I’d say make it shorter. I already make the decision before I download the app (Participant One).*

*Normally I would skip such a video, especially if it takes 3 min. I would directly click on “go to the login screen” … (*Interviewer: *How long should the video be?) Well, about one minute. I do understand that all that information does not fit in a minute. There is a whole section about privacy which is very good. But I think, if I want to know exactly how that is organized, I’ll look it up. I just assume that that’s okay (Participant Eleven).*

However, others felt that the video had a suitable length. One participant mentioned that the video could be extended to include more information, which was currently only available in the supplementary texts.

*Fine, not too long. Nice voice-over. The video is short enough. I would stop it if it would take any longer, 10 min would be far too long. For such a study I would watch the complete 3 min, this is important after all (Participant Eighteen).*

*The video was a bit short, it could have been a little longer. There could have been more in it. I miss a number of things that are part of the text but are not so clear in the video. I think the video should be 4 or 5 min (Participant Twenty-nine).*

#### Opinions on the app’s content and preferences for types of information sources

Participants expressed their satisfaction with the fact that they were given the choice to receive the information in a video or in writing. The participants who watched the animated video said the information was conveyed in a clear and understandable manner. Some said that they remembered the information about the study better through the images in the film. The cartoonist style of the video was most often found to be attractive and fitting to the message. Participants in particular responded well to the narrator of the video, who, according to them, spoke clearly and explained the study at the right pace. A few participants felt that the visual effects in the video were at times too chaotic, which distracted them from the spoken information.

*The video explains a lot, so I didn’t feel the need to read much more… But I think it’s good that you get information in both ways (Participant Six).*

*The video is clear. I didn’t really look at the text much. I’m not very good at reading. The video works much better for me than reading a piece (Participant Fifteen).*

*The style of the video is nice. Children with all those different colours, really fits Amsterdam. It is also nice for children themselves later on: the researcher with the coat is recognizable, children who are sad and children who are happy… well done! (Participant Sixteen)*

*Nice clear voice. I do think the video is quite busy. You look here and there and then something pops up again: it was visually busy. I think if you have a clear voice trying to explain things then maybe it should go by topic…. Of course it’s about one topic, but that you don’t have that many of those pop up things (Participant Twenty-three).*

The few participants who read the script version of the video noted that the text as a whole was too long and contained sentences that were too lengthy. They also mentioned that this text was a bit boring. More positive responses were given to the supplementary text blocks with detailed background information. These were seen as understandable texts and the length was considered more suitable.

On the script version of the video:

*Yes, it was clear. … I was in doubt… the sentences were long, stretched long across the screen (Participant Seventeen).*

*There was also a lot of text. In the end I thought I should have watched the video instead…* (On the supplementary text blocks) *This is a bit nicer and shorter. Then you are also able to choose. And such a summary with four dots. Great, yes, I like that. That’s what I would expect if I clicked text (Participant Eleven).*

#### Opinions on the identification procedure

All but one of the participants were able to complete the 2 step identification procedure by a log-in with the provided usernames and passwords of the test account and subsequently filling out the date of birth of the test case children. Due to a technical issue that delayed data retrieval in the identification process, several participants asked if something had gone wrong because they couldn’t proceed right away. The first time this occurred the observers were unfamiliar with this issue and aborted the test. As for later cases, the participants said they missed a notification that data retrieval was still in progress.

In the following interview, participants gave mixed reactions to the identification procedure. Although all said to be familiar with DigiD as an identification tool for health insurance or tax return purposes, many mentioned they were surprised to see that they needed DigiD to participate in research. A few mentioned they had some hesitation in using their personal DigiD for such a purpose. In their view, this contradicted the fact that we use their children’s data anonymously for research. One participant said that the mandatory DigiD registration was a reason for him to refrain from participating. Conversely, there were also participants who saw the use of DigiD as something positive. For them, DigiD felt like a trusted, confidential and secure way to give consent.

*The notion that it’s all so private, that notion fades a bit. Things are well recorded after all… If you are going to log in with your DigiD, how private is the data? (Participant Five).*

*Yes, that is normal these days, isn’t it? the data is quite personal. I would not just log in with some code. Better to use DigiD. DigiD is really confidential. Everyone needs a DigiD (Participant Six).*

When inquiring whether participants would be able to log in with their own personal username and password, more than half of them answered that they knew this by heart. Others said they didn’t know their username and password, but kept them at home.

*In general, I always forget my password. I never remember it. I don’t know how that works for other people. Coincidentally, I know my DigiD at the moment. But if I don’t know it at this moment, it does not have my priority to look it up or request a new password. I don’t know how that usually goes… (Participant Twenty-eight).*

*No, I do not know my password by heart. I have it at home, in a folder. I do not think people just know this by heart. Where do you use DigiD for? Only for important stuff. Things that you do at home (Participant Fifteen).*

#### Participants’ ability to recall study information

Most participants said they found themselves sufficiently informed by the app to be able to make a choice about their participation. The participants who felt they were not well enough informed said they missed information on what types of data were used and for which specific research questions we would use this. When we asked them to tell us something about the study, nearly all participants remembered that the cohort is a study about the health of zero till 18-year-old children that grow up in Amsterdam. Some participants, however, had some misconceptions. For instance, one parent thought the study was conducted to give parents personal advice about the healthy upbringing of their child. When asked what participants should do now that they had given consent, most had understood that they would receive health questionnaires as part of the study. Yet, nearly a quarter of the participants did not recall this aspect. Considering the types of care data that are collected for the study, the majority of participants could correctly name them. Others gave both correct and incorrect examples of data. A few mentioned they had not seen this information or gave an incorrect answer based on a false assumption. About data collection, almost half of the participants remembered that the health data is first encrypted, after which it is securely stored to be used anonymously for research. A third of the participants could only name one aspect about the data collection. A couple of participants could not recall any details about the data collection.

## Discussion

This study explored whether an informed consent app can contribute to a comprehensive and practical process for obtaining informed parental consents in a socio-economically and culturally diverse population. In a qualitative study we tested a prototype of the informed consent app designed for a large-scale population-based cohort study. To get a sense of parents’ views on the usability, look & feel and content of the informed consent app, we asked them to comment on the app while using it. In addition, we conducted a semi-structured interview to further elaborate on their points of view. A second objective was to evaluate how well our app functions as a tool to inform participants about the study. This was done by asking questions about the study’s information that was provided in the app, right after our participants gave their test consent.

### Participants’ ability and satisfaction of using an informed consent app

Participants were able to successfully follow the steps to consent to participate in the Sarphati Cohort. In general, they found the app to be well designed and for the most part easy to use. Although some pages of the app were identified as less user-friendly due to non-intuitive button placement or malfunctioning input fields, none of these issues prevented participants from registering their consent in the app. As most participants had a clear preference to receive the information in either video or in writing, many were happy they could choose between both. Most participants used the animation video as a primary source of information, a smaller group preferred to read the texts. Reactions on the video were predominantly positive. Many found that the video explained the study in a clear and understandable manner. Participants’ opinions on the various texts in the app varied more. Where the text version of the video was considered a bit long and unattractive, the extra optional background texts were experienced as more accessible due to their shorter length.

### Challenges when using an identification system

An interesting finding in our study was the opposing views on the identification system used to verify that person is eligible to participate and is allowed to give parental consent. Some participants believed that having to use an identification system that is known to give access to highly private personal data contradicted the message that researchers can never see their child’s personal health care data. However, others were more reassured that such a well-known system was used to securely give permission. Such privacy-related concerns might be generalisable to other countries that have similar systems. In particular for countries within Europe, where an EU-wide electronic identification framework is now being developed that would enable the mutual recognition of national electronic identification systems (eID) across borders [22]. These trust issues relate to findings of Simon et al. who reported on the trust barriers of minorities in the US towards using electronic informed consents and privacy and confidentiality concerns of rural participants [19]. Concerns about privacy and confidentiality were also reported by Hentschel and colleagues to be one of the dominant factors in pregnant women’s willingness to share electronic health records of themselves and their infant [23]. In order to create more trust among parents in the digital informed consent procedure, it might be helpful to invest in personal face to face contact. This is also suggested by both Anderson and Yusof, who point out that while new technology has a great potential to improve informed consent procedures, they can never fully replace human interaction. Human interaction should remain a central aspect when obtaining informed consent [17, 24].

### How well do participants get informed by a digital consent app?

As a second objective of this study we assessed the performance of the app as an information tool by asking 4 open-ended information retention questions on the provided information of the study. We asked questions about what the study entails, what participation means for parents, what data is used and how collected data are being handled and used. Overall, we found that most participants had a general idea of the study. At the same time, we also observed that some participants were less well informed as they couldn’t give a satisfactory answer to one or more questions. Multiple factors may have influenced the ability of participants to recall the provided information. Among them the amount of time participants spent with the app. We found large differences between participants. Some spent less than half the time viewing the information materials than others did. Where most of the participants watched the entire video intently, occasionally combing it with reading of the supplementary texts, others chose to skip large parts of the video and proceeded directly to the consent page. Afterwards they explained they found the 3-minute video too long. Findings of participants skipping information are similar to issues reported in paper consent forms [25]. For example, McNutt and colleagues found that, after verbally describing the study, half of their participants would read a consent form in 30 s or less before signing. Likewise, Baren and colleagues found that only 13% of all participants who want to participate spent more than 2 min reading their consent form. Even though we have tried to get our audience more engaged, by offering them information on our study in multiple forms, it may be unavoidable that there will always be individuals who consent without being fully aware of what they consent to. Nevertheless, for the vast majority, properly informing potential participants seems to lie in striking a balance between tailoring the content to the needs of the individual while not making too many concessions on the quality and completeness of the information.

### Strengths and limitations

This study has some limitations. One of them is that we recruited participants through convenience sampling. People who tend to participate in a study like ours are generally more interested in participating in research. Our sample of participants may less accurately reflect the total population eligible to join the Sarphati Cohort. Such a selection bias could cause our results to be overly optimistic in terms of participants being able to give consent. Also since the app was only available in Dutch at the time of this study we could only include participants who understood Dutch sufficiently. Therefore, our results may not be generalisable to ethnic minority groups with low Dutch language proficiency. Looking further at our sample of participants, the male-female ratio in our study is 1 to 5. While there were no obvious differences between men and women, this may be due to the small size of the male group. Whether this underrepresentation of males should be seen as a significant limitation to our results is not immediately clear. In fact, the 1 to 5 ratio may accurately reflect the parent that will initially be approached to participate in our study. Additionally, there are some other limitations in this study regarding the assessment about the app’s potential as an information tool. It remains debatable whether, based on the information retention questions, we have indeed acquired a valid measure on the app’s ability to inform. We deliberately choose to use open ended questions, as multiple choice questions are less cognitively demanding and portray the ability to recognize rather than remember [26]. However, open-ended questions give room for interpretation. The assessment of whether a participant had correctly recalled the information could often not simply be captured in a dichotomous yes or no. Also our assessment was subjected to several confounding factors. For instance, not all participants considered the topics that were questioned important for deciding whether or not to give consent. Some felt that some information was too detailed and should be optional for those interested. As a result, they may have paid less attention to the parts of the video or texts in which this was explained. Another confounder that might affect the person’s ability to recall information was the presence of two investigators during the sessions. Whether this helped, as the participant studied the materials more carefully, or whether this worked against them due to the stress of a testing situation is not clear.

A strength of this study is that it was designed to resemble a real life setting. We therefore usually held the tests at the participants’ homes. We also deliberately did not ask our participants to carefully read and/or view all information, but to simply use the app as they normally would. As such, we tried to obtain a more realistic picture of the situation in which parents are likely to use the app in the future. Our findings show that not all participants take the expected route to inform themselves through the app, seems that we have achieved this at least to some degree. Future studies in which both digital and non-digital methods can be compared should shed more light on how digital consent procedures affect participation rates, and whether they lead to better informed participants.

## Conclusion

The findings of this study suggest that a well-designed digital informed consent app has the potential to inform participants about research and enable them to give consent. We found that participants experienced no significant problems using an app to give their digital consent. The ability of digital formats allows to broadcast information in both video and writing, which was greatly appreciated by our participants and should be optimally exploited. However, our research also identified some shortcomings of digital informed consents, including trust barriers towards the identification procedure and individuals who consented while not fully familiarizing themselves with all the provided materials. This made us aware that in order to properly inform potential participants in the diverse population of Amsterdam we cannot rely solely on a digital app as a panacea. In order to get our target population to visit our consent app, we need to already get them somewhat interested in participating in the study. A broader recruitment strategy was drafted where non-digital content such as posters, flyers and brochures and personal contact both on site and remotely in the form of a helpdesk play an important role in supporting the digital consent app. Especially regarding the privacy related concerns, that some of our participants expressed, investing in personal contact seems crucial in the pursuit of truly inclusive recruitment. Such an approach might be especially effective for groups that often seem to be underrepresented in research. Digital consent procedures can provide a good solution to efficiently enrol participants in large population-based studies, but don’t seem to eliminate the need for non-digital human interaction.

### Footnote: summary of what has happened to the app since the study

Testing the app with Amsterdam parents led to the identification of a number of issues, both in terms of user-friendliness and content. A list of desired changes to the app was compiled by the research team and shared with the app designer. This resulted in several adjustments to improve usability, such as button placement and styling. In terms of content, several textual changes were made to better inform users about the study and consent procedure, including adjusting the text explaining why logging in with DigiD is necessary and adding a text to the consent page that summarizes in four points what participation in the cohort entails. Subsequently the app was translated to English, French, Spanish, Turkish and German. The consent app and the website version of the app was successfully launched by the end of 2018 (www.sarphati-app.nl). To ensure a successful start of the inclusion of the cohort, the team was fully deployed for recruitment in the first year (i.e. 2019). In 2020 some improvements were made to the app and a Moroccan Arabic video was included in the app to better inform potential participants. The article was originally intended to be submitted in 2020, but then the COVID pandemic hit. The capacity of our team, since we work for the Public Health Service of Amsterdam, was partly focused on combatting the pandemic, while the remainder was being deployed for the continuation of data collection and recruitment of the cohort in an adapted form. Writing this article was therefore postponed and could not be picked up again until after the pandemic.

### Electronic supplementary material

Below is the link to the electronic supplementary material.

Supplementary Material 1

## Data Availability

The datasets analysed during the current study are available from the corresponding author on reasonable request.

## Abbreviations

DigiD: Digital Identity

## References

[1] Gemeente Amsterdam and Onderzoek en Statistiek., “Bevolking naar nationaliteiten, 1 januari 2019–2021,” 2022. Accessed: Oct. 19, 2022. [Online]. Available: https://data.amsterdam.nl/datasets/bx_HyaOipADV-Q/stand-van-de-bevolking-amsterdam/?term=Stand+van+de+bevolking+Amsterdam.

[2] Gemeente Amsterdam and Onderzoek en Statistiek., “Bevolking van 15–74 naar hoogst afgeronde opleidingsniveau,” 2020. Accessed: Oct. 21, 2022. [Online]. Available: https://onderzoek.amsterdam.nl/dataset/kerncijfers-onderwijs-amsterdam.

[3] Michon L, Meester F, Rubingh S, Verhaar S, de Jong I. “Nederlandstalige laaggeletterden in Amsterdam Onderzoek, Informatie en Statistiek,” 2021. Accessed: Oct. 21, 2022. [Online]. Available: https://onderzoek.amsterdam.nl/publicatie/nederlandstalige-laaggeletterden-in-amsterdam.

[4] Perrenoud B, Velonaki VS, Bodenmann P, Ramelet AS (2015). The effectiveness of health literacy interventions on the informed consent process of health care users: a systematic review protocol. JBI Database of Systematic Reviews and Implementation Reports.

[5] Foe G, Larson EL. Reading level and comprehension of research consent forms: an integrative review. J Empir Res Hum Res Ethics. Feb. 2016;11(1):31–46. 10.1177/1556264616637483. 10.1177/1556264616637483 27106889

[6] Falagas ME, Korbila IP, Giannopoulou KP, Kondilis BK, Peppas G. Informed consent: how much and what do patients understand? Am J Surg. Sep. 2009;198:420–35. 10.1016/j.amjsurg.2009.02.010. no. 3. 10.1016/j.amjsurg.2009.02.010 19716887

[7] Ittenbach RF, Senft EC, Huang G, Corsmo JJ, Sieber JE. “Readability and understanding of informed consent among participants with low incomes: A preliminary report,” *Journal of Empirical Research on Human Research Ethics*, vol. 10, no. 5, pp. 444–448, Dec. 2015, 10.1177/1556264615615006. 10.1177/1556264615615006 26564942

[8] Montalvo W, Larson E. “Participant Comprehension of Research for Which They Volunteer: A Systematic Review,” *Journal of Nursing Scholarship*, vol. 46, no. 6, pp. 423–431, Nov. 2014, 10.1111/jnu.12097. 10.1111/jnu.12097 25130209

[9] Cheung KL, ten Klooster PM, Smit C, de Vries H, Pieterse ME. The impact of non-response bias due to sampling in public health studies: a comparison of voluntary versus mandatory recruitment in a Dutch national survey on adolescent health. BMC Public Health. 2017;17(1). 10.1186/s12889-017-4189-8. 10.1186/s12889-017-4189-8 PMC5363011 28330465

[10] Rothwell E, et al. A randomized controlled trial of an electronic informed consent process. J Empir Res Hum Res Ethics. Dec. 2014;9(5):1–7. 10.1177/1556264614552627. 10.1177/1556264614552627 PMC5847281 25747685

[11] Rowbotham MC, Astin J, Greene K, Cummings SR. Interactive informed consent: Randomized comparison with Paper consents. PLoS ONE. Mar. 2013;8(3). 10.1371/journal.pone.0058603. 10.1371/journal.pone.0058603 PMC3590180 23484041

[12] Warriner AH, et al. A pragmatic randomized trial comparing tablet computer informed consent to traditional paper-based methods for an osteoporosis study. Contemp Clin Trials Commun. Aug. 2016;3:32–8. 10.1016/j.conctc.2016.02.003. 10.1016/j.conctc.2016.02.003 PMC5935867 29736454

[13] Flory J, Emanuel E. Interventions to improve research participants’ understanding in informed consent for Research. JAMA. Oct. 2004;292(13):1593. 10.1001/jama.292.13.1593. 10.1001/jama.292.13.1593 15467062

[14] Nishimura A, Carey J, Erwin PJ, Tilburt JC, Murad MH, McCormick JB. Improving understanding in the research informed consent process: a systematic review of 54 interventions tested in randomized control trials. BMC Med Ethics. 2013;14(1). 10.1186/1472-6939-14-28. 10.1186/1472-6939-14-28 PMC3733934 23879694

[15] Gesualdo F, et al. Digital tools in the informed consent process: a systematic review. BMC Med Ethics. Dec. 2021;22(1). 10.1186/s12910-021-00585-8. 10.1186/s12910-021-00585-8 PMC7913441 33639926

[16] Moe-Byrne T, et al. Does digital, multimedia information increase recruitment and retention in a children’s wrist fracture treatment trial, and what do people think of it? A randomised controlled study within a trial (SWAT). BMJ Open. Jul. 2022;12(7). 10.1136/bmjopen-2021-057508. 10.1136/bmjopen-2021-057508 PMC9280884 35831055

[17] Anderson EE, Newman SB, Matthews AK. “Improving informed consent: Stakeholder views,” *AJOB Empir Bioeth*, vol. 8, no. 3, pp. 178–188, Jul. 2017, 10.1080/23294515.2017.1362488. 10.1080/23294515.2017.1362488 PMC5749407 28949896

[18] Mahnke AN et al. “A rural community’s involvement in the design and usability testing of a computer-based informed consent process for the personalized medicine research project,” *Am J Med Genet A*, vol. 164, no. 1, pp. 129–140, Jan. 2014, 10.1002/ajmg.a.36220. 10.1002/ajmg.a.36220 PMC4145717 24273095

[19] Simon CM, Schartz HA, Rosenthal GE, Eisenstein EL, Klein DW. Perspectives on electronic informed consent from patients underrepresented in Research in the United States: a Focus Group Study. J Empir Res Hum Res Ethics. Oct. 2018;13(4):338–48. 10.1177/1556264618773883. 10.1177/1556264618773883 29790410

[20] Ministry of the Interior and Kingdom Relations., “What is DigiD?” 2023. https://www.digid.nl/en/what-is-digid (accessed Sep. 18, 2023).

[21] Bailey J. “First steps in qualitative data analysis: Transcribing,” *Fam Pract*, vol. 25, no. 2, pp. 127–131, Apr. 2008, 10.1093/fampra/cmn003. 10.1093/fampra/cmn003 18304975

[22] Commission E. “eID Offers digital services capable of electronically identifying users from all across Europe.,” Mar. 07, 2021. https://ec.europa.eu/digital-building-blocks/wikis/display/DIGITAL/eID#:~:text=eID%20is%20a%20set%20of,services%20from%20other%20European%20countries. (accessed Sep. 07, 2023).

[23] Hentschel A et al. Perspectives of pregnant and breastfeeding women on participating in longitudinal mother-baby studies involving electronic health records: qualitative study, JMIR Pediatr Parent, vol. 4, no. 1, Jan. 2021, doi: 10.2196/23842.M. Y. P. M. 10.2196/23842 PMC8080167 33666558

[24] Yusof CH, Teo, Ng CJ. Electronic informed consent criteria for research ethics review: a scoping review. BMC Med Ethics. Nov. 2022;23(1):117. 10.1186/s12910-022-00849-x. 10.1186/s12910-022-00849-x PMC9682656 36414962

[25] Ripley KR, Hance MA, Kerr SA, Brewer LE, Conlon KE. “Uninformed Consent? The Effect of Participant Characteristics and Delivery Format on Informed Consent,” *Ethics Behav*, vol. 28, no. 7, pp. 517–543, Oct. 2018, 10.1080/10508422.2018.1456926.

[26] Connor Desai S, Reimers S. Comparing the use of open and closed questions for web-based measures of the continued-influence effect. Behav Res Methods. 2019;51(3). 10.3758/s13428-018-1066-z. 10.3758/s13428-018-1066-z PMC6538818 29943224

