# Supplementary material for: Developing a digital informed consent app: opportunities and challenges of a new format to inform and obtain consent in public health research
Source: BMC Med Ethics. 2023 Nov 8;24:97. doi: 10.1186/s12910-023-00974-1 (PMC10634039; doi:10.1186/s12910-023-00974-1)
Supplement: Supplementary file 1 — Supplementary Material 1 [file 12910_2023_974_MOESM1_ESM.docx]

**Script test app & semi-structured interview:**

**Assignment:**

Go through the app as if we weren't there. Try to put yourself in the shoes of the parent of these test-case child(ren) as much as possible.

- If a parent indicates that they do not wish to participate in the Sarphati Cohort and clicks no 🡪 What is the reason for not giving consent at this time?
  - Because we want to practically test the app today, I would like to ask you to give permission for this test.

**Retention questions after using IC-app**

1. Can you tell me in your own words what you think the Sarphati cohort entails?
2. What can you expect as a parent now you have registered your child for the Sarphati Cohort?
3. What kind of data about your child is used for research?
4. How do we use your child's data for research?

**Interview on user experience**

1. What do you think of the app in general?

- Do you find the Sarphati Cohort app easy to use?
  - If not, what could be better?
- Does the style of the video and app appeal to you as a parent?
  - If not, what could be better?
- What do you think of the comprehensibility and clarity of the video?
  - and of the text?

1. Did you encounter any practical difficulties when using the app?
2. Do you think that you have been informed well enough by the app to make a choice to participate in the Sarphati Cohort?

- Do you think there should have been other information in the video?
- Could you easily find the necessary (additional) information in the app?

1. Based on the information provided, do you have any reasons why you would hesitate to give your consent for this study?
2. What do you think of the necessary login with DigiD?

- Was it clear from the text why the DigiD login is necessary?
- Would the DigiD login procedure with your own DigiD be a problem?
  - Do you know your own DigiD by heart?
  - Do you have your DigiD somewhere at home?
  - Would you like to receive additional help, if so, in what form?
  - Preference digital versus paper consent?

1. What do you think of the Sarphati Cohort in general?

- After all you now know about the Sarphati Cohort, would you also register your own children for the Sarphati Cohort?

1. How and when would you prefer to be informed about the Sarphati Cohort?
2. At what location would you prefer to use the app to provide consent?
